# Mānuka oil based ECMT-154 versus vehicle control for the topical treatment of eczema: study protocol for a randomised controlled trial in community pharmacies in Aotearoa New Zealand

**DOI:** 10.1186/s12906-024-04358-9

**Published:** 2024-01-29

**Authors:** Gabrielle Shortt, Nicholas Shortt, Georgina Bird, Kyley Kerse, Nico Lieffering, Alexander Martin, Allie Eathorne, Bianca Black, Bob Kim, Marius Rademaker, Louise Reiche, Selwyn Te Paa, Suki Harding, Mike Armour, Alex Semprini

**Affiliations:** 1 Medical Research Institute of New Zealand, Wellington, Aotearoa New Zealand; 2 Victoria University of Wellington, Wellington, Aotearoa New Zealand; 3 NICM Health Research Institute, Western Sydney University, Penrith, Australia; 4 University of Otago, Wellington, Aotearoa New Zealand; 5 Waikato Clinical Campus, University of Auckland, Hamilton, Aotearoa New Zealand; 6 Anderson’s Exchange Pharmacy, Dunedin, Aotearoa New Zealand; 7 New Zealand Dermatological Society Inc, Palmerston North, Aotearoa New Zealand; 8 New Zealand Dermatology Research Trust, Palmerston North, Aotearoa New Zealand; 9 Manuka Bioscience Ltd, Auckland, Aotearoa New Zealand

**Keywords:** Eczema, Emollient, Botanical therapy, Pharmacy-based research network, Decentralised

## Abstract

**Background:**

Eczema is a chronic, relapsing skin condition commonly managed by emollients and topical corticosteroids. Prevalence of use and demand for effective botanical therapies for eczema is high worldwide, however, clinical evidence of benefit is limited for many currently available botanical treatment options. Robustly-designed and adequately powered randomised controlled trials (RCTs) are essential to determine evidence of clinical benefit. This protocol describes an RCT that aims to investigate whether a mānuka oil based emollient cream, containing 2% ECMT-154, is a safe and effective topical treatment for moderate to severe eczema.

**Methods:**

This multicentre, single-blind, parallel-group, randomised controlled trial aims to recruit 118 participants from community pharmacies in Aotearoa New Zealand. Participants will be randomised 1:1 to receive topical cream with 2% ECMT-154 or vehicle control, and will apply assigned treatment twice daily to affected areas for six weeks. The primary outcome is improvement in subjective symptoms, assessed by change in POEM score. Secondary outcomes include change in objective symptoms assessed by SCORAD (part B), PO-SCORAD, DLQI, and treatment acceptability assessed by TSQM II and NRS.

**Discussion:**

Recruitment through community pharmacies commenced in January 2022 and follow up will be completed by mid-2023. This study aims to collect acceptability and efficacy data of mānuka oil based ECMT-154 for the treatment of eczema. If efficacy is demonstrated, this topical may provide an option for a novel emollient treatment. The community-based design of the trial is anticipated to provide a generalisable result.

**Ethics and dissemination:**

Ethics approval was obtained from Central Health and Disability Ethics Committee (reference: 2021 EXP 11490). Findings of the study will be disseminated to study participants, published in peer-reviewed journal and presented at scientific conferences.

**Trial registration:**

Australian New Zealand Clinical Trials Registry (ANZCTR) ACTRN12621001096842. Registered on August 18, 2021 (https://www.anzctr.org.au/Trial/Registration/TrialReview.aspx?id=382412&isReview=true).

**Protocol version:**

2.1 (Dated 18/05/2022).

## Background

Eczema is a common, chronic or recurrent inflammatory skin condition, characterised by acute flares of pruritic lesions [1, 2]. Although prevalence is highest in children, for many it can persist into, or even develop in, adulthood [3–6]. Aotearoa New Zealand has a high prevalence of disease, with Māori and Pacific populations having a greater disease burden than New Zealand Europeans [7, 8]. Eczema represents a significant individual and societal financial burden, and contributes to a strain on healthcare resources [9–11]. Additionally, patient quality of life is significantly affected due to persistent itch, pain, disturbance of sleep, and emotional distress [12, 13].

Eczema is caused by complex interactions of genetics, environmental factors, and immune activation [14, 15]. The primary mechanisms for the pathophysiology include skin barrier dysfunction and immune dysregulation [16, 17]. Filaggrin is a key protein in epidermal barrier function, preventing water loss and entry of allergens and infectious organisms [18]. Two common loss-of-function mutations in the gene encoding Filaggrin can lead to an epidermal barrier defect, and have been strongly linked to the development of eczema [19, 20]. This reduction of epidermal integrity can cause allergen and microbe introduction, leading to activation of the immune system and inflammatory response [21]. The immune response in patients with eczema is associated with CD4^+^ T cells (Th) and upregulated expression of Th2 and Th22 cytokines, resulting in chronic inflammation [22–24].

Skin colonisation by *Staphylococcus aureus (S. aureus)* is common among patients with eczema, at a carriage rate much higher than the general population [25–27] *S. aureus* is associated with disease pathogenesis, and symptom flares which can result in loss of skin microbiota diversity, further allowing *S. aureus* to dominate which can lead to significant secondary infections [28–30].

There is currently no cure for eczema, treatment instead focuses on symptom management, including identification and avoidance of irritants that exacerbate symptoms [31–33]. The primary strategy for symptom management includes the regular use of emollients to maintain and restore skin barrier function [34]. Topical corticosteroids or calcineurin inhibitors effectively reduce inflammation and are often used to treat symptom flares, or used prophylactically to maintain disease control [32, 33, 35]. However, up to 80% of eczema patients report fears around topical corticosteroids use, with concerns of side effects leading to low treatment adherence, a major contributing factor in treatment failure, and consequent poor disease control [36–38].

Prevalence of use and demand for plant-derived botanical therapies for eczema is high worldwide, [39, 40] however high quality clinical evidence for the efficacy of such products is limited. ECMT-154 is a formula comprising β-triketone rich mānuka oil and palmarosa oil, both of which demonstrate anti-inflammatory and antimicrobial activity [41–44]. In vitro, mānuka oil reduces lipopolysaccharide-induced release of inflammatory cytokine TNF-α [41]. The geraniol component of palmarosa oil has also demonstrated inhibition of various inflammatory pathways in vitro and in vivo [42, 45–47]. β-triketone rich mānuka oil and geraniol are also both effective agents against gram-positive bacteria, such as *S. aureus* [44, 48–50]. The anti-inflammatory effects may be effective in relieving eczema symptoms when lesions develop while anti-staphylococcal activity may be beneficial by reducing eczema severity and lower the risk of secondary infections. This randomised controlled trial (RCT) is designed to investigate whether in vitro evidence may translate to clinical benefit, through reduction of symptoms in patients with eczema.

This study aims to assess efficacy of a novel, non-steroidal topical emollient cream containing 2% ECMT-154 via an RCT in community pharmacies.

## Methods and analysis

### Study design

This study is a parallel-group, superiority, assessor-blinded RCT assessing the efficacy of 2% ECMT-154 compared with vehicle control in the topical treatment of moderate to severe eczema in adults. The trial protocol has been developed in accordance with Standard Protocol Items: Recommendations for Interventional Trials (SPIRIT) guidelines [51].

### Trial setting and recruitment

This study will be conducted using the Medical Research Institute of New Zealand (MRINZ) Pharmacy Research Network (PRN), an established network of over 80 research trained community pharmacists in Aotearoa New Zealand overseen centrally by researchers at the MRINZ [52, 53]. Between 10–15 pharmacies will recruit participants and undertake study related procedures. Pharmacies were selected based on previous recruitment success and capacity to complete study procedures. Adults presenting to a PRN pharmacy seeking advice for eczema will be screened for eligibility. Advertising within participating pharmacies and on mainstream or social media will be used as a recruitment tool.

### Screening and selection

An anonymous pre-screening survey will be available for potential participants to self-screen against eligibility criteria (Table 1). At the pharmacy participants will be screened using a predefined statement to determine suitability for the study, followed by anonymised assessment of Patient Oriented Eczema Measure (POEM) score [54]. If participants decline participation or do not meet the inclusion criteria of POEM score (≥ 8 to ≤ 24), no further information will be collected. Moderate to severe eczema is required to prevent a floor effect, and increase the likelihood of detecting a significant change in POEM score. Willing participants provide written informed consent, and undergo assessment of eligibility.

**Table 1:** Eligibility criteria

Inclusion criteria	• Participant is willing and able to provide written informed consent • Participant is aged between 18 and 65 years, inclusive • Participant reported, doctors diagnosis of eczema • Patient has a representative eczema lesion, located below the clavicle that is in an area they are comfortable having photographed • Patient has a POEM score of ‘moderate to severe eczema’ (8 to 24) • Participant is willing to stop all moisturisers and other skin barrier cream or emulsion during the treatment period and replace it with the investigational product assigned in this trial. Usual facial regimens and application of sunscreen is permitted • Participant is willing to replace their body wash/soap with aqueous cream as supplied at enrolment • Participant is able to attend a follow up visit six weeks after they enrol in the study. This will take place at a participating pharmacy or via telephone call if required due to COVID restrictions or unanticipated inability to attend in person • Participant is willing and able to comply with the study and comply with all study procedures
Exclusion criteria	• Current requirement for antibiotics or corticosteroids for the treatment of any condition (with the exception of inhaled and intranasal corticosteroids) • Use of topical and/or oral antibiotics, corticosteroids, or antihistamines within the last two weeks (with the exception of inhaled and intranasal corticosteroids) • Use of immunomodulatory medications taken for eczema within the past four weeks • Cutaneous mycotic or bacterial disease requiring a topical or systemic therapy • Other skin condition which may affect the assessment of eczema • History of allergy or hypersensitivity to study treatment ingredients • Participation in a clinical study involving an investigational product during the last three months • Participant is pregnant/breastfeeding or planning to become pregnant during the study • Cold/flu like symptoms, fever, or unexplained shortness of breath in the past 14 days • Any other condition which, at the investigators’ discretion, is believed may present a safety risk or impact upon the ability of the participant to complete the study

### Randomisation and masking

Participants are randomised 1:1 to receive a 2% ECMT-154 cream or vehicle control. Participants will be block randomised, block size four, and randomisation will be stratified according to site. A computer-generated randomisation number sequence will be created by the study statistician. Randomisation of participants takes place at the pharmacy, electronically within REDCap. Participants will be randomised by pharmacy investigators who have no access to the randomisation schedule. Manufacturing and labelling of interventional treatments will be conducted by an external compounding pharmacist, licensed to complete such activities and assessed according to Good Manufacturing Practice guidelines. Active treatment and vehicle control will be labelled as “Treatment G” and “Treatment H” in non-descript packaging for the purpose of blinding. Study investigators at the pharmacies and MRINZ will be blinded. Participants will not be told if they receive the active or control cream, however due to the distinctive smell of ECMT-154 compared to control, participants are assumed to be unblinded therefore this study will conservatively be classified as single blind rather than double blind.

### Interventional treatments

Study intervention, and vehicle control are expected to provide emollient benefits from ingredients in the base cream, comprised of polyethylene glycol (300 and 3350), water, white soft paraffin, stearyl alcohol, propylene glycol, and sodium lauryl sulphate. Treatments are identical in formulation, with the exception of 2% ECMT-154 added to interventional cream. Participants will be dispensed two 500 gram tubs of study treatment for liberal application to affected areas twice daily, morning and night, for six weeks. In addition, three 500 gram tubs of aqueous cream (Boucher, India) will be supplied to replace participants usual soap and body wash. Adherence to randomised treatment will be collected in weekly diaries with participants asked how many times they used their treatment each day over the past week.

### Participant timeline

**Fig. 1:**
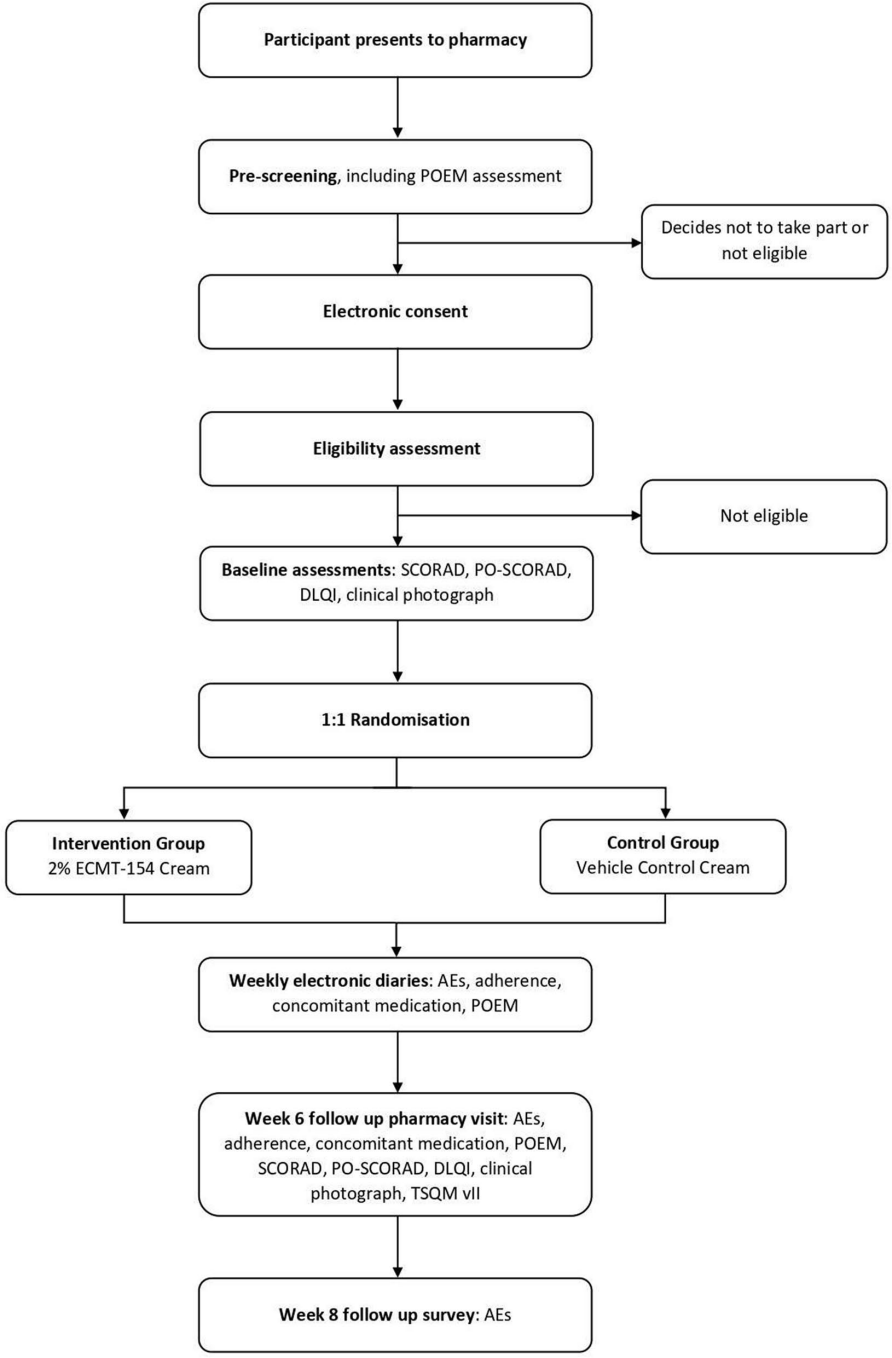
Study flow diagram

Upon presentation to the pharmacy, consent, demographic data, eligibility, and baseline data are collected, followed by a clinical photograph of a representative eczema lesion, chosen by the participant. The participant is randomised, and the appropriate treatment is dispensed by the study pharmacist along with the aqueous cream. Over the six-week interventional period, participants complete a weekly digital diary to report POEM, treatment adherence, adverse events, and use of concomitant medications (Table 2). Participants return to the pharmacy for a second face to face visit at week six including a second clinical photograph of the representative lesion. A final electronic follow up survey is completed at week eight to collect any adverse events following the cessation of study treatment. The study flow is depicted in Fig. 1*.*

**Table 2:** Summary of study procedures

	Visit 1	Treatment Period					Visit 2	Follow Up Survey
Day	**0**	**7**	**14**	**21**	**28**	**35**	**42 (+ 5)**	**14 days post Visit 2 (+ 5)**
Eligibility screen	X							
Informed consent	X							
Demographic data collection	X							
POEM	X	X	X	X	X	X	X	
PO-SCORAD	X						X	
SCORAD (Part B)	X						X	
DLQI	X						X	
Eczema photo	X						X	
Randomisation	X							
Dispense medication	X							
Electronic study diary		X	X	X	X	X		
Treatment compliance		X	X	X	X	X	X	
Adverse event collection		X	X	X	X	X	X	X
TSQM Version II							X	
Acceptability NRS							X

### Retention

Participants are reimbursed $200 (NZD) in recognition of travel and time. Reminders for weekly study diary completion are sent to non-responders once daily for five days, after which the diary window is closed.

### Permitted and prohibited concomitant treatment

No other eczema treatments or moisturisers will be permitted during the trial. Participants can continue with all other prescribed medication. Concomitant medication usage will be collected in the weekly participant diaries, which ask asked if participants have used any medications other than their study treatment in the last week, prompts them to leave details and MRINZ investigators will contact the participant directly to complete concomitant medication forms based on C-DASH standards. It is recognised that minor exacerbations of eczema are expected, therefore the usage of topical corticosteroids habitually used by the participant, for a duration of less than or equal to seven days, is permitted without requiring withdrawal from the study. The requirement for escalated treatment will be recorded and compared between treatment groups.

### Data capture

All study related data, including informed consent, will be captured using e-source via REDCap-based case report forms [55]. REDCap is a secure, United States Health Insurance Portability and Accountability Act 1996 (HIPAA) compliant web-based application designed to support data capture for research studies. CDASH-compliant structures are used to collect standard data types required for this study. Data is entered into tablet devices at pharmacy sites. Online study diaries and questionnaires completed by study participants are accessed via a link unique to each participant for each time point. The usage of electronic data capture allows for data validation using data entry ranges, required data fields, and logic checks. Study data is stored on secure password protected servers and only accessible by trial staff and independent study auditors, investigators will retain access to the final trial dataset.

### Monitoring

A study monitoring plan, independent from the Sponsor, is in place to ensure all study conduct complies with International Council for Harmonisation of Technical Requirements for Registration of Pharmaceuticals for Human Use, Good Clinical Practice and New Zealand ethical guidelines. All substantial protocol deviations and violations will be reported to the New Zealand ethics committee as per approval requirements.

### Outcome measures

#### Primary

Primary outcome is severity assessed by subjective symptoms over six weeks, measured by change in POEM scores, adjusted for baseline. The POEM tool assesses severity of eczema by capturing self-reported frequency of symptoms over the previous seven days [54]. Scores can range from 0 to 28, with higher scores indicating a higher severity of disease. The following bandings have been established: Clear or almost clear (0–2); Mild eczema (3–7); Moderate eczema (8–16); Severe eczema (17–24); Very severe eczema (25–28).

#### Secondary

Secondary outcome measures include participants who had a four or greater reduction in POEM score (termed ‘responders’ [56]); the difference in POEM scores analysed per protocol; the difference in POEM scores; the difference in Patient Oriented SCORing Atopic Dermatitis (PO-SCORAD) [57]; the difference in pharmacist-assessed SCORing Atopic Dermatitis (SCORAD) score (part B only) [58]; withdrawals due to the exacerbation of eczema; the proportion of treatment escalation between groups; the difference in Dermatology Life Quality Index (DLQI) [59, 60]; participant acceptance of the treatment measured by Treatment Satisfaction Questionnaire for Medication (TSQM) Version II [61]; Numerical Rating Scale (NRS) of the acceptability of the treatment; the proportion of cutaneous and systemic events between the treatment groups; the difference in SCORAD scores between blinded pharmacist and blinded dermatologist; and the difference in SCORAD scores between pharmacists. For full schedule of interventions and assessments see Table 2*.*

#### Statistical analysis

Primary outcome analysis, and analysis of secondary POEM, PO-SCORAD, and DLQI outcomes will be by ANCOVA with baseline POEM score as a continuous covariate, and randomised treatment as a categorical variable of interest. Analysis of proportions will be by estimation of relative risk and associated confidence intervals. Participant acceptability will be assessed by t-test, or if normality assumptions are strongly violated by Mann–Whitney test with Hodges-Lehmann estimator of location. Comparison of SCORAD scores between pharmacists and between pharmacist and dermatologist will be by linear mixed model with participant treated as a random effect.

Analysis will be separated by intention-to-treat (ITT) and per protocol set (PPS), with ITT as the primary analysis. ITT population is all participants randomised. PPS is all participants with at least 80% completed data, including primary outcome with no significant protocol deviations determined to influence POEM score. For inclusion in PPS, participants should adhere to treatment instructions, measured by > 80% daily adherence. Missing outcome data will not be imputed. No interim analyses are planned and SAS will be used for analysis.

#### Power calculation

The sample size calculation is based on a minimal clinically important difference (MCID) of 3.4 [62] and pooled standard deviation (SD) of 6.0 for the change in POEM score [52]. With 80% power, and 5% two-sided alpha, each treatment group would require 50 participants. Allowing for 15% withdrawal and loss to follow up, 118 participants total are required for this study.

#### Participant safety

A study specific safety plan is in place for the trial. AEs will be identified by participant diaries, at pharmacy visits, or by ad-hoc contact from participants to the study team. Participants are prompted in their weekly diary with an open question asking if they have experienced any changes in their health or if they have experienced worsening eczema in the last week in order to capture all a wide-variety of adverse events. Any affirmative response results in contact from central MRINZ investigator for formal AE collection. All AEs are reviewed by the study doctor and coordinating team within 24 hours. An Independent Data and Safety Monitoring Committee (DSMC) is appointed to monitor all adverse events on a three-monthly basis, and will be informed of any SAEs within 72 hours. If safety concerns are expressed by the DSMC, unblinded study data may be made available to them on request. The DSMC may advise the study should be halted or terminated in the case of significant concerns for the safety of participants. AEs will be reported every six months to Medsafe, the New Zealand Medicine and Medical Devices Safety Authority. Indemnity insurance is in place for study sponsor for claims resulting from trial participation.

## Discussion

There is increasing demand worldwide for effective botanical therapies for eczema as an adjunct to conventional treatment. This community-based randomised controlled trial will determine if in vitro evidence for mānuka oil based ECMT-154 translates to clinical benefit.

This decentralised study will use direct electronic data capture, allowing real-time remote monitoring of study visits. Weekly follow up of participants through electronic diaries allows the study team to maintain safety oversight throughout participation in the study.

Community pharmacies are well positioned to facilitate trial participation for individuals with that may otherwise be faced with barriers accessing clinical trials [63, 64]. It is anticipated this study design will provide a more generalisable result than if recruitment was conducted at a single study site, which could otherwise introduce bias on ethnicity or socioeconomic status.

A secondary outcome of this study will assess pharmacist capability for SCORAD scoring, a tool typically used by trained dermatologists. Training will be provided to pharmacists and SCORAD scores will be compared to remote dermatologist assessment. Results may help inform appropriate selection of outcome assessments for future research conducted in community pharmacies.

In conclusion, this trial will provide high quality safety and efficacy data on mānuka oil based ECMT-154 for the topical treatment of eczema. If efficacy is demonstrated, this novel topical treatment may provide a steroid-sparing emollient option for individuals with eczema.

### Trial status

At the time of submission, participant recruitment began in January 2022. Enrolment is anticipated to continue through to mid-2023. Current protocol version 2.1 dated 18th May 2022.

## Data Availability

Study results will be submitted for publication to an appropriate academic peer-reviewed journal, and will be presented at relevant conferences. Results will be shared with participant and all recruiting pharmacy sites. Findings will be reported in accordance with the CONSORT (Consolidated Standards of Reporting Trials) statement.

## Abbreviations

AE: Adverse Event
DLQI: Dermatology Life Quality Index
DSMC: Data and Safety Monitoring Committee
HDEC: Health and Disability Ethics Committee
ITT: Intention-to-treat
MCID: Minimal Clinically Important Difference
NRS: Numerical Rating Scale
PO-SCORAD: Patient-Oriented SCORing Atopic Dermatitis
POEM: Patient-Oriented Eczema Measure
PPS: Per Protocol Set
RCT: Randomised Controlled Trial
SAE: Serious Adverse Event
SCORAD: SCORing Atopic Dermatitis
SCOTT: Standing Committee on Therapeutic Trials
TSQM: Treatment Satisfaction Questionnaire for Medication

## References

[1] Nutten S (2015). Atopic Dermatitis: Global Epidemiology and Risk Factors. ANM.

[2] Johansson SGO, Bieber T, Dahl R, Friedmann PS, Lanier BQ, Lockey RF (2004). Revised nomenclature for allergy for global use: Report of the Nomenclature Review Committee of the World Allergy Organization, October 2003. J Allergy Clin Immunol.

[3] Abuabara K, Yu AM, Okhovat JP, Allen IE, Langan SM (2018). The prevalence of atopic dermatitis beyond childhood: A systematic review and meta-analysis of longitudinal studies. Allergy.

[4] Margolis JS, Abuabara K, Bilker W, Hoffstad O, Margolis DJ (2014). Persistence of mild to moderate atopic dermatitis. JAMA Dermatol.

[5] Barbarot S, Auziere S, Gadkari A, Girolomoni G, Puig L, Simpson EL (2018). Epidemiology of atopic dermatitis in adults: Results from an international survey. Allergy.

[6] Silverberg JI, Hanifin JM (2013). Adult eczema prevalence and associations with asthma and other health and demographic factors: a US population-based study. J Allergy Clin Immunol.

[7] Clayton T, Asher MI, Crane J, Ellwood P, Mackay R, Mitchell EA (2013). Time trends, ethnicity and risk factors for eczema in New Zealand children: ISAAC Phase Three. Asia Pac Allergy.

[8] Odhiambo JA, Williams HC, Clayton TO, Robertson CF, Asher MI (2009). ISAAC Phase Three Study Group. Global variations in prevalence of eczema symptoms in children from ISAAC Phase Three. J Allergy Clin Immunol.

[9] Ring J, Zink A, Arents BWM, Seitz IA, Mensing U, Schielein MC (2019). Atopic eczema: burden of disease and individual suffering - results from a large EU study in adults. J Eur Acad Dermatol Venereol.

[10] Carroll CL, Balkrishnan R, Feldman SR, Fleischer AB, Manuel JC (2005). The burden of atopic dermatitis: impact on the patient, family, and society. Pediatr Dermatol.

[11] Zink AGS, Arents B, Fink-Wagner A, Seitz IA, Mensing U, Wettemann N (2019). Out-of-pocket Costs for Individuals with Atopic Eczema: A Cross-sectional Study in Nine European Countries. Acta Derm Venereol.

[12] Silverberg JI, Gelfand JM, Margolis DJ, Boguniewicz M, Fonacier L, Grayson MH (2018). Patient burden and quality of life in atopic dermatitis in US adults: A population-based cross-sectional study. Ann Allergy Asthma Immunol.

[13] Holm EA, Wulf HC, Stegmann H, Jemec GBE (2006). Life quality assessment among patients with atopic eczema. Br J Dermatol.

[14] Kantor R, Silverberg JI (2017). Environmental risk factors and their role in the management of atopic dermatitis. Expert Rev Clin Immunol.

[15] De Benedetto A, Agnihothri R, McGirt LY, Bankova LG, Beck LA (2009). Atopic Dermatitis: A Disease Caused by Innate Immune Defects?. J Investig Dermatol.

[16] Bos JD, Wierenga EA, Sillevis Smitt JH, van der Heijden FL, Kapsenberg ML (1992). Immune dysregulation in atopic eczema. Arch Dermatol.

[17] Novak N, Leung DYM (2011). Advances in atopic dermatitis. Curr Opin Immunol.

[18] McAleer MA, Irvine AD (2013). The multifunctional role of filaggrin in allergic skin disease. J Allergy Clin Immunol.

[19] O’Regan GM, Sandilands A, McLean WHI, Irvine AD (2008). Filaggrin in atopic dermatitis. J Allergy Clin Immunol.

[20] Palmer CNA, Irvine AD, Terron-Kwiatkowski A, Zhao Y, Liao H, Lee SP (2006). Common loss-of-function variants of the epidermal barrier protein filaggrin are a major predisposing factor for atopic dermatitis. Nat Genet.

[21] Cork MJ, Danby SG, Vasilopoulos Y, Hadgraft J, Lane ME, Moustafa M (2009). Epidermal Barrier Dysfunction in Atopic Dermatitis. J Investig Dermatol.

[22] Leung DYM (2013). New Insights into Atopic Dermatitis: Role of Skin Barrier and Immune Dysregulation. Allergol Int.

[23] Gittler JK, Shemer A, Suárez-Fariñas M, Fuentes-Duculan J, Gulewicz KJ, Wang CQF (2012). Progressive activation of Th2/Th22 cytokines and selective epidermal proteins characterizes acute and chronic atopic dermatitis. J Allergy Clin Immunol.

[24] Dubin C, Del Duca E, Guttman-Yassky E (2021). The IL-4, IL-13 and IL-31 pathways in atopic dermatitis. Expert Rev Clin Immunol.

[25] Higaki S, Morohashi M, Yamagishi T, Hasegawa Y (1999). Comparative study of staphylococci from the skin of atopic dermatitis patients and from healthy subjects. Int J Dermatol.

[26] Guzik TJ, Bzowska M, Kasprowicz A, Czerniawska-Mysik G, Wójcik K, Szmyd D (2005). Persistent skin colonization with *Staphylococcus aureus* in atopic dermatitis: relationship to clinical and immunological parameters. Clin Exp Allergy.

[27] Leyden JJ, Marples RR, Kligman AM (1974). *Staphylococcus aureus* in the lesions of atopic dermatitis. Br J Dermatol.

[28] Geoghegan JA, Irvine AD, Foster TJ (2018). Staphylococcus aureus and Atopic Dermatitis: A Complex and Evolving Relationship. Trends Microbiol.

[29] Tauber M, Balica S, Hsu CY, Jean-Decoster C, Lauze C, Redoules D (2016). *Staphylococcus aureus* density on lesional and nonlesional skin is strongly associated with disease severity in atopic dermatitis. J Allergy Clin Immunol.

[30] Totté JEE, van der Feltz WT, Hennekam M, van Belkum A, van Zuuren EJ, Pasmans SGMA (2016). Prevalence and odds of *Staphylococcus aureus* carriage in atopic dermatitis: a systematic review and meta-analysis. Br J Dermatol.

[31] Simpson EL (2010). Atopic dermatitis: a review of topical treatment options. Curr Med Res Opin.

[32] Chong M, Fonacier L (2016). Treatment of Eczema: Corticosteroids and Beyond. Clin Rev Allergy Immunol.

[33] Weidinger S, Novak N, Kiel C (2016). Atopic dermatitis. Lancet.

[34] van Zuuren EJ, Fedorowicz Z, Christensen R, Lavrijsen A, Arents BW (2017). Emollients and moisturisers for eczema. Cochrane Database Syst Rev.

[35] Lee JH, Son SW, Cho SH. A Comprehensive Review of the Treatment of Atopic Eczema. Allergy Asthma Immunol Res. 2016;8(3):181–90. 10.4168/aair.2016.8.3.181 PMC4773205 26922927

[36] Li AW, Yin ES, Antaya RJ (2017). Topical Corticosteroid Phobia in Atopic Dermatitis: A Systematic Review. JAMA Dermatol.

[37] Lee JY, Her Y, Kim CW, Kim SS (2015). Topical Corticosteroid Phobia among Parents of Children with Atopic Eczema in Korea. Ann Dermatol.

[38] Aubert-Wastiaux H, Moret L, Le Rhun A, Fontenoy AM, Nguyen JM, Leux C (2011). Topical corticosteroid phobia in atopic dermatitis: a study of its nature, origins and frequency. Br J Dermatol.

[39] See A, Teo B, Kwan R, Lim R, Lee J, Tang MBY (2011). Use of complementary and alternative medicine among dermatology outpatients in Singapore. Australas J Dermatol.

[40] Lu CL, Liu XH, Stub T, Kristoffersen AE, Liang SB, Wang X (2018). Complementary and alternative medicine for treatment of atopic eczema in children under 14 years old: a systematic review and meta-analysis of randomized controlled trials. BMC Complement Altern Med.

[41] Chen CC, Yan SH, Yen MY, Wu PF, Liao WT, Huang TS (2016). Investigations of kanuka and manuka essential oils for *in vitro* treatment of disease and cellular inflammation caused by infectious microorganisms. J Microbiol Immunol Infect.

[42] Wang J, Su B, Zhu H, Chen C, Zhao G (2016). Protective effect of geraniol inhibits inflammatory response, oxidative stress and apoptosis in traumatic injury of the spinal cord through modulation of NF-κB and p38 MAPK. Exp Ther Med.

[43] Song CY, Nam EH, Park SH, Hwang CY (2013). In vitro efficacy of the essential oil from Leptospermum scoparium (manuka) on antimicrobial susceptibility and biofilm formation in Staphylococcus pseudintermedius isolates from dogs. Vet Dermatol.

[44] Prashar A, Hili P, Veness RG, Evans CS (2003). Antimicrobial action of palmarosa oil (Cymbopogon martinii) on Saccharomyces cerevisiae. Phytochemistry.

[45] Mączka W, Wińska K, Grabarczyk M. One Hundred Faces of Geraniol. Molecules. 2020;25(14):3303. 10.3390/molecules25143303 PMC7397177 32708169

[46] Ye CJ, Li SA, Zhang Y, Lee WH (2019). Geraniol targets KV1.3 ion channel and exhibits anti-inflammatory activity *in vitro* and *in vivo*. Fitoterapia.

[47] Huang Y, Yang XL, Ni YH, Xu ZM (2018). Geraniol suppresses proinflammatory mediators in phorbol 12-myristate 13-acetate with A23187-induced HMC-1 cells. Drug Des Devel Ther.

[48] Bard M, Albrecht MR, Gupta N, Guynn CJ, Stillwell W (1988). Geraniol interferes with membrane functions in strains of Candida and Saccharomyces. Lipids.

[49] Christoph F, Kaulfers PM, Stahl-Biskup E (2000). A comparative study of the in vitro antimicrobial activity of tea tree oils s.l. with special reference to the activity of beta-triketones. Planta Med.

[50] van Klink JW, Larsen L, Perry NB, Weavers RT, Cook GM, Bremer PJ (2005). Triketones active against antibiotic-resistant bacteria: synthesis, structure-activity relationships, and mode of action. Bioorg Med Chem.

[51] Chan AW, Tetzlaff JM, Altman DG, Laupacis A, Gøtzsche PC, Krleža-Jerić K (2013). SPIRIT 2013 statement: defining standard protocol items for clinical trials. Ann Intern Med.

[52] Shortt N, Martin A, Kerse K, Shortt G, Vakalalabure I, Barker L (2022). Efficacy of a 3% Kānuka oil cream for the treatment of moderate-to-severe eczema: A single blind randomised vehicle-controlled trial. eClinicalMedicine.

[53] Semprini A, Singer J, Braithwaite I, Shortt N, Thayabaran D, McConnell M (2019). Kanuka honey versus aciclovir for the topical treatment of herpes simplex labialis: a randomised controlled trial. BMJ Open.

[54] Spuls PI, Gerbens LAA, Simpson E, Apfelbacher CJ, Chalmers JR, Thomas KS (2017). Patient-Oriented Eczema Measure (POEM), a core instrument to measure symptoms in clinical trials: a Harmonising Outcome Measures for Eczema (HOME) statement. Br J Dermatol.

[55] Harris PA, Taylor R, Thielke R, Payne J, Gonzalez N, Conde JG (2009). Research electronic data capture (REDCap)-A metadata-driven methodology and workflow process for providing translational research informatics support. J Biomed Inform.

[56] Howells L, Ratib S, Chalmers JR, Bradshaw L, Thomas KS, CLOTHES trial team (2018). How should minimally important change scores for the Patient-Oriented Eczema Measure be interpreted? A validation using varied methods. Br J Dermatol.

[57] Stalder JF, Barbarot S, Wollenberg A, Holm EA, De Raeve L, Seidenari S (2011). Patient-Oriented SCORAD (PO-SCORAD): a new self-assessment scale in atopic dermatitis validated in Europe. Allergy.

[58] Kunz B, Oranje AP, Labrèze L, Stalder JF, Ring J, Taïeb A (1997). Clinical validation and guidelines for the SCORAD index: consensus report of the European Task Force on Atopic Dermatitis. Dermatology.

[59] Finlay AY, Khan GK (1994). Dermatology Life Quality Index (DLQI)–a simple practical measure for routine clinical use. Clin Exp Dermatol.

[60] Ali FM, Johns N, Finlay AY, Salek MS, Piguet V (2017). Comparison of the paper-based and electronic versions of the Dermatology Life Quality Index: evidence of equivalence. Br J Dermatol.

[61] Atkinson MJ, Kumar R, Cappelleri JC, Hass SL (2005). Hierarchical construct validity of the treatment satisfaction questionnaire for medication (TSQM version II) among outpatient pharmacy consumers. Value Health.

[62] Schram ME, Spuls PI, Leeflang MMG, Lindeboom R, Bos JD, Schmitt J (2012). EASI, (objective) SCORAD and POEM for atopic eczema: responsiveness and minimal clinically important difference. Allergy.

[63] Unger JM, Gralow JR, Albain KS, Ramsey SD, Hershman DL (2016). Patient Income Level and Cancer Clinical Trial Participation: A Prospective Survey Study. JAMA Oncol.

[64] Price KN, Lyons AB, Hamzavi IH, Hsiao JL, Shi VY (2021). Facilitating Clinical Trials Participation of Low Socioeconomic Status Patients. DRM.

[65] Medicines Act 1981. New Zealand Legislation; 2021 Dec. Report No.: 118. https://www.legislation.govt.nz/act/public/1981/0118/latest/whole.html#DLM55429. Cited 2022 Jun 20.

